# Validation of Optimization Methods for Sensory Characteristics Using Rate-All-That-Apply and Intensity Scales: A Case Study of Apple Juice

**DOI:** 10.3390/foods13172853

**Published:** 2024-09-08

**Authors:** Yoojin Jeong, Han Sub Kwak, Manyoel Lim, Young Jun Kim, Youngseung Lee

**Affiliations:** 1 Department of Food Science and Nutrition, Dankook University, Cheonan 31116, Republic of Korea; tnsdldbwls@naver.com; 2 Food Processing Research Group, Korea Food Research Institute, Wanju-gun 55465, Republic of Korea; hskwak@kfri.re.kr (H.S.K.); manyoell@kfri.re.kr (M.L.); 3 KFRI School, University of Science and Technology, Wanju-gun 55465, Republic of Korea; 4 Department of Food and Biotechnology, Korea University, Sejong 30019, Republic of Korea; yk46@korea.ac.kr

**Keywords:** apple juice, descriptive analysis, external preference mapping, intensity scale, rate all that apply

## Abstract

Preference mapping (PM), which integrates consumer and descriptive analysis (DA) data to identify attributes that drive consumer liking, is widely employed for product optimization. However, a limited group of trained panelists cannot fully represent the diverse consumer population or reliably predict market acceptance. Consequently, numerous studies have explored consumer-based methodologies as potential replacements for DA; however, their efficacy for product optimization remains limited. Therefore, this study was conducted to explore the potential of optimizing products using two consumer-based profiling techniques as alternatives to DA in external preference mapping (EPM). Overall, 8 trained panelists profiled 12 sensory attributes of 7 commercial apple juices, whereas 160 consumers assessed the same attributes using a 5-point rate-all-that-apply (RATA) scale and a 10 cm intensity scale (IS). Danzart’s response surface ideal modeling was employed to identify optimal products using DA, RATA, and IS through barycenter calculations, focusing on three products from the original consumer test located around the group ideal point. Overall, the ideal products of the group and their sensory characteristics were successfully identified using DA, RATA, and IS. Regarding sensory intensities, high concordance was observed between DA and RATA (Rv = 0.92) and between DA and IS (Rv = 0.91). Overall liking and preference scores for products mixed at the optimal ratio for each method showed no significant differences in preference among the ideal products identified using DA, RATA, and IS. This study suggests that both RATA and IS are viable alternatives to DA in EPM for identifying ideal sensory profiles.

## 1. Introduction

Preference mapping (PM) is a multivariate analysis technique employed to identify consumer preferences for products. It is primarily used to optimize products through the integration of consumer preference and product sensory data [1]. This can be categorized into external and internal types. Internal PM involves applying principal component analysis (PCA) to individual consumer preference data to create a consumer preference space, followed by regressing mechanical or descriptive analysis (DA) data onto that space [2]. Conversely, external PM (EPM) creates a product space by analyzing mechanical or DA data to define product differences and subsequently regresses individual consumer preference data onto that space [2]. The resulting preference map can be used to identify characteristics that positively or negatively influence consumer preferences and locate the products of competitors when developing new products [1,3].

Specifically, response surface ideal point mapping (RSIPM), devised by Danzart, is used for EPM. RSIPM considers the area above the average preference value of all consumers as the acceptable preference area for each consumer [4]. Once these acceptable areas are established, a density plot can be generated by overlapping all consumer areas, and the densest point can be considered as the ideal product. Subsequently, the sensory profile of the ideal product can be obtained using the barycenter method based on its location on the map [5].

Apples, one of the most consumed fruits in Korea, are the fruit of deciduous apple trees belonging to the Rosaceae family. They are rich in minerals, vitamins, antioxidants dietary fiber, and polyphenol compounds [6,7]. These nutrients facilitate bowel movements and help prevent degenerative diseases such as diabetes, high blood pressure, and arteriosclerosis [8]. Apples have been traditionally consumed raw; however, recent dietary trends have led to their processing and consumption in the form of juice, jam, and dairy products. Over 90% of processed apple products are in the form of juices and drinks [6,7]. In the case of apple juice, most raw materials can be sourced domestically in Korea and maintained in high quality, rendering it a highly viable fruit drink option. Research on the sensory characteristics and consumers’ hedonic perception of apple juice has been previously reported. Okayasu et al. [9] identified the sensory characteristics of 16 commercial apple juice samples (4 clarified and 12 unclarified) using evaluations from 140 consumers and 10 trained panelists. Their findings indicated that apple juice with moderately enhanced fresh and green aromas, along with reduced sourness and astringency, was more preferred by consumers. Włodarska et al. [10] studied the sensory and hedonic perception of eight differently processed clear and cloudy commercial apple juices. They found that consumers showed the highest preference for freshly squeezed juices, while cloudy juices not from concentrate and clear juices made from concentrate were significantly less liked.

In sensory science, DA has been extensively employed to ensure food product quality and drive innovation within the food industry [11]. However, DA panelists may not accurately capture the hedonic perception of a product, as their training emphasizes specific standards, which may not consistently align with individual consumer preferences. Furthermore, a limited group of trained panelists cannot fully represent the diverse consumer population or reliably predict market acceptance [11]. Therefore, rapid profiling methods based on consumer perception have gained popularity in the food industry as alternatives to traditional descriptive methods. Consumer-based profiling methods require no training, optimize time and resources, and provide useful insights [12]. Notable examples include check all that apply (CATA), rate all that apply (RATA), free listing, and intensity scales (ISs). Several studies have assessed the potential of consumer-based profiling methods as alternatives to conventional DA. However, studies on extending these methods to PM for product optimization remain limited.

Therefore, this study aimed to identify the ideal sensory profile of apple juice by replacing DA in EPM with two representative consumer-based profiling methods. In addition, the potential of these consumer-based profiling methods to serve as viable alternatives to DA was assessed, along with an additional preference test to assess the validity of the proposed ideal products.

## 2. Materials and Methods

### 2.1. Samples

The most popular commercially available apple juices in Korea based on sales were initially selected. Juices with similar flavor profiles or brand duplication were excluded, resulting in a final inclusion of seven commercial apple juices. These samples represented a broad range of product quality owing to variations in their raw materials or flavors added. All seven samples used were subjected to DA and consumer tests, including RATA, IS, and a validation test. Detailed product descriptions are provided in Table 1.

### 2.2. Descriptive Analysis

For the DA, eight panelists, comprising two males and six females in their 20s, were selected based on their high accuracy in the basic taste, flavor, and mouthfeel screening tests. They also demonstrated the capability to differentiate between samples based on the established standard samples. Prior to training, the selected panelists underwent an orientation on the concept and scale of the spectrum method (Sensory Spectrum Inc., Chatham, NJ, USA). Training sessions were conducted weekly over 3 months, totaling 16 h. After the completion of basic training, panelists were assigned to assess the attributes of apple juice to create a sensory lexicon. During the evaluation sessions, the panelists were asked to freely identify characteristics while tasting apple juices. Characteristics that achieved unanimous agreement were determined through group meetings, with discussions continuing until all panelists reached a consensus regarding the types and intensities of standard samples for the given sensory attributes. The resulting lexicon (Table 2), comprising 12 characteristics, included 2 characteristics related to appearance, 3 related to aroma, 5 related to flavor, and 2 related to mouthfeel. The panelists evaluated these characteristics using a 16-point numerical scale, consistent with their training.

Samples were presented to the panelists in duplicate using a complete randomized block design. A 10 min break was allocated between these replications to allow the panelists to refresh their palates. Additionally, water and spitting cups were provided for mouth rinsing between samples. This study was approved by the Institutional Review Board of Dankook University (IRB No. DKU 2023-03-061-004).

### 2.3. Consumers

Consumers were recruited through school advertisements and online platforms featuring a QR code, which allowed interested individuals to participate in an electronic questionnaire. This questionnaire collected information such as age, sex, the frequency and time of apple juice consumption, and purchase factors. A total of 240 consumers were included in the study, evenly divided into 3 groups of 80 for each testing method, as detailed subsequently. All procedures adhered to the guidelines of the Institutional Review Board of Dankook University (IRB No. DKU 2023-03-061-004), and participants provided informed consent for the utilization of personal information before evaluation. Consumers assessed their overall liking (OL) of the product using a 9-point verbal hedonic scale (1 = dislike extremely to 9 = like extremely). The test followed a sequential monadic design, with sample presentation orders balanced at each serving position using the Williams design. Prior to evaluation, the seven apple juices were refrigerated, and 40 mL of each were dispensed into plastic cups labeled with a unique 3-digit code for blind tasting.

### 2.4. Rate All That Apply

Overall, 80 consumers (67 females and 13 males aged 20–40 years) participated in the RATA analysis. The same set of 12 characteristics used in DA were employed for the RATA analysis. To minimize serving order and numerical biases, samples were presented following a Williams Latin Square design, and random codes were assigned to the samples. During evaluation, consumers first rated the OL of each sample, followed by a RATA analysis. Consumers were allowed to freely select the characteristics they deemed suitable for describing the samples, subsequently rating the intensity of these characteristics on a 5-point scale (1 = “low”, 3 = “medium”, 5 = “high”).

### 2.5. Intensity Scale

Eighty participants (52 females and 28 males aged 20–50 years) were asked to complete the IS questionnaire. Similar to the RATA analysis, these participants assessed 12 characteristics previously identified through DA. To minimize potential numerical bias, samples were labeled with random codes and presented in a randomized order using a Williams Latin Square design. During evaluation, consumers first rated the OL of each sample, followed by an IS questionnaire. Consumers evaluated the intensity of each characteristic using a 10 cm unstructured line scale anchored with “low” at the left and “high” at the right [13]. In this scale, 0 cm denotes “not present,” 5 cm indicates “medium”, and 10 cm represents “very strong”.

### 2.6. Product Optimization

Product optimization was performed using the RSIPM method, an EPM approach devised by Danzart. RSIPM creates a multidimensional sensory space for products through PCA using sensory data [4]. This space, known as the sensory space, accommodates regression of consumer preference data. To determine the consumer acceptable area, Danzart suggests using the mean preference score derived from all consumers across the products. Therefore, the consumer acceptable area is identified as the area that surpasses the mean preference value of all consumers. Finally, the acceptable areas of all consumers are overlapped, and the densest point is identified as the ideal product [5]. The barycenter method can be used to determine the sensory profile of the identified ideal product. Consumer OL data for optimization were collected from 160 consumers, with 80 each participating in the RATA and IS assessments. The sensory data of the products, subsequently used to create the sensory space, were obtained from DA, RATA, and IS evaluations.

### 2.7. Validation Test

An additional 80 consumers (69 females and 11 males aged 20–40 years) participated in the validation test. This test was conducted to determine the differences in OL among the products optimized through DA and those optimized using two the consumer-based profiling methods. The validation test included three ideal products using the RSIPM method, which incorporated sensory data from DA and the two consumer-based profiling methods (i.e., RATA and IS). In addition to the ideal products, products highly preferred in previous consumer tests (*n* = 160) were included. The OL and preference rankings of the products were evaluated using a 9-point scale and a ranking method.

### 2.8. Data Analysis

Analysis of variance (ANOVA) was employed to assess significant differences between products in terms of the intensity of sensory attributes obtained from DA and the two consumer-based profiling methods. The RSIPM method, used for product optimization, was implemented using Visual Basic for Applications within Excel to validate ideal product configurations and calculate optimal ratios. The sensory profiles of the ideal products obtained through the three different optimization methods were compared by calculating correlation coefficients. The similarity between the sensory characteristics of the ideal products and the seven samples was assessed using the Rv coefficient to examine consistency across methods [14]. The OL data obtained through the validation test were analyzed using ANOVA to assess potential differences in preference among the ideal products optimized through DA and the two consumer-based profiling methods (α = 0.05). Additionally, a Friedman test was performed to assess potential significant differences in preference rankings among the products tested (α = 0.05).

## 3. Results and Discussion

### 3.1. Descriptive Analysis

The DA results for the 12 characteristics of the seven apple juices are presented in Table 3. ANOVA revealed significant differences for all 12 characteristics among the samples (*p* < 0.05). The panelists identified strong “yellowness” in P7, which is 100% apple juice. Based on the product information, P7 is produced using a blend of various apple types, encompassing “Newtown Pippin”, “Granny Smith”, “Gala”, “Honeycrisp”, “Fuji”, and “Golden Delicious”. Among them, “Honeycrisp”, “Gala”, and “Fuji” apples are known for their orange/reddish color on a yellow base, whereas “Golden Delicious” apples have a golden color [15,16]. Accordingly, the high “yellowness” of P7 can be attributed to the orange-yellow color of its raw materials.

High levels of “turbidity” were observed in P2, P4, P5, and P6, whereas the other samples exhibited low “turbidity” intensities. These differences are likely attributed to the variations in the solid content levels of the apple juices [17]. The high “turbidity” in P4 and P6 can be attributed to their elevated apple solid contents, specifically 12.0% and 12.5%, respectively (calculated from Table 1). Pinelo et al. [18] reported that the interaction of proteins with pectin in apples can affect juice turbidity. Therefore, the high “turbidity” in P2 may result from its apple paste, pectin, and skim milk powder content. Additionally, the relatively high turbidity in P5 is likely attributed to its inclusion of apple puree [19].

Although the exact processing information of the samples used in this study is unknown, given that they were sourced commercially, the samples with high turbidity may have been produced using direct pressing of the mash without additional enzyme treatment. This method usually results in juice containing pulp particles dispersed in a serum composed of macromolecules, such as pectin and proteins, which contribute to the cloudy appearance of apple juice [19]. The highest intensities of “sour aroma” and “sourness” were detected in P3, P4, and P5, which contained vitamin C in their raw materials. Additionally, citric acid was present in P3 and P4, as shown in Table 1.

Metallic notes can be perceived in apple juice [20] as apples naturally contain trace amounts of metal elements such as iron and copper [21]. These metal elements can create a metallic sensation under acidic conditions. Apple juice is typically subjected to acidic conditions; therefore, attributes such as metallic aroma can be elicited [22]. High levels of “metallic aroma” were identified in P5 and P6. In a study by Hashizume et al. [23], panelists described cloudy apple juice exposed to light as having a metallic sensation. Moreover, Umekawa [24] detected a metallic off-flavor in grape juice stored in PET bottles. Given that P5 and P6 were retailed in plastic bottles, both the packaging material and exposure to light may have contributed to the high “metallic aroma” detected in them. The highest intensities of “apple aroma” and “apple flavor” were detected in P7, which was 100% apple juice. High levels of “artificial flavor” were detected in P1 and P2, likely attributed to the addition of natural sweeteners present in them. P1 contained enzymatically modified stevia, whereas P2 contained enzymatically modified stevia and thaumatin. Waldrop and Ross [25] reported that consumers expressed unpleasantness for stevia owing to its association with an artificial flavor. Moreover, Han et al. [26] reported that stevia sweeteners have an astringent aftertaste and a residual bitter taste. These findings may explain the strong identification of an “artificial flavor” in P1 and P2, which contain a stevia-based sweetener.

P7 exhibited the highest intensities of “sweetness” and “honey flavor.” Although specific processing details are unavailable, one of the apple cultivars used in P7 is believed to be “Fuji”, possibly making up the largest proportion of the blend. Notably, the “Fuji” cultivar is known for its heightened sweetness and honey flavor [15]. During apple juice processing, a caramelization reaction occurs during heat treatment [27], resulting in the formation of various compounds from sugar degradation. These compounds significantly contribute to the flavor, aroma, and color of caramel [28]. Therefore, the caramel flavor may have contributed to the honey flavor in P7.

“Astringency”, associated with “sourness”, was highest in P4, which also exhibited the highest “sourness” intensity among the samples [29]. Lawless et al. [29] demonstrated that the astringency of certain acids could directly indicate their acidic properties, independent of the hydrogen bonding mechanisms involved in the interaction between tannins and salivary proteins.

### 3.2. Rate All That Apply

RATA was assessed using the same sensory attributes used for DA. Significant differences were observed for all characteristics among the samples (Table 4).

Consumers observed a pronounced “yellowness” in both P4 and P7, consistent with DA findings. The turbidity and color of apple juices are influenced by polyphenol oxidase (PPO) [30]. Juices directly pressed with the mash tend to exhibit high turbidity owing to the dispersal of its pulp particles in a serum containing macromolecules, including pectin or proteins [19]. Therefore, pressed apple juices without special treatments may undergo enzymatic browning due to PPO activity [31]. Although specific processing information is unavailable, P4, which exhibited the highest “turbidity”, may have undergone a color change attributable to the browning reaction. The attributes of “sour aroma” and “sourness” were most pronounced in P3, P4, and P5, all of which contain vitamin C (Table 4), aligning with DA results. “Metallic aroma” also showed results similar to those of DA, being most strongly perceived in P5 and P6. Among the samples, P1, which contained high-fructose corn syrup and enzymatically modified stevia, exhibited the highest “sweetness” intensity. Consistent with DA findings, “artificial flavor” was prominent in P1 and P2, whereas “honey flavor” was strong in P7.

However, unlike DA, no significant difference in “apple flavor” was observed among the samples, except for P2. P2 contains sweeteners such as thaumatin and enzymatically modified stevia, with thaumatin being 2000 to 10,000 times sweeter than sugar [32]. Pimentel et al. [33] reported that clarified apple juice with added sucralose exhibited diminished apple flavor due to a masking effect from the intense sweetness compared to that of pure juice, suggesting that the sweetness may have influenced the apple flavor of the product. Given that sucralose, which is approximately 600 times sweeter than sugar, exhibited these effects, the sweeteners in P2 may have also had a similar masking effect. “Astringency” was strongly identified in P4, which also had a high intensity of “sourness”, consistent with previous research showing an association between astringency and sourness [29]. Overall, RATA results exhibited a pattern similar to DA results, confirming that consumers could discriminate between apple juices to a degree equivalent to that of trained descriptive panelists.

### 3.3. Intensity Scale

The IS method was also performed using the same 12 attributes used for DA and RATA. Significant differences were observed for all 12 characteristics (Table 5).

Unlike DA, consumers identified “yellowness” most strongly in P4. Similar to the RATA results, this observation may be attributed to enzymatic browning caused by PPO in apples [31]. Regarding aroma-related characteristics, such as sour, metallic, and apple aroma, the intensity trends among samples were consistent with those observed in DA. P2 exhibited the lowest intensity of “apple flavor”, possibly because of the masking effect from strong sweetness, as observed in the RATA results. Consistent with DA findings, “turbidity”, “sourness”, and “astringency” were strongly identified in P4. “Artificial flavor” was prominently perceived in P1 and P2, whereas “honey flavor” was strongly noted in P7.

### 3.4. Response Surface Ideal Point Mapping

#### 3.4.1. DA Data

**Figure 1:**
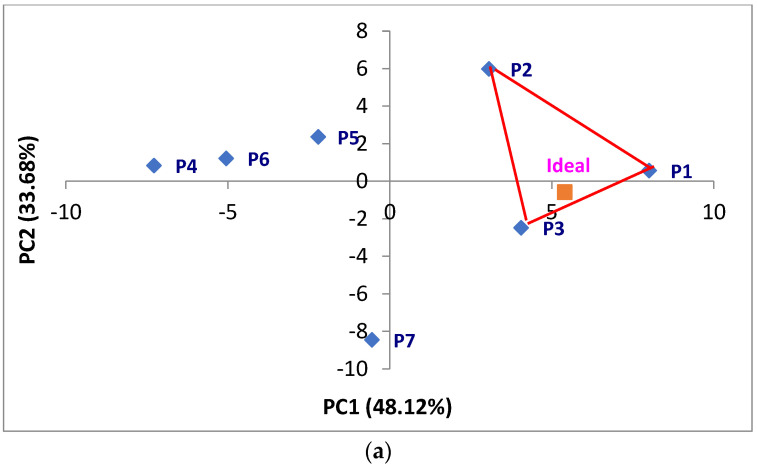

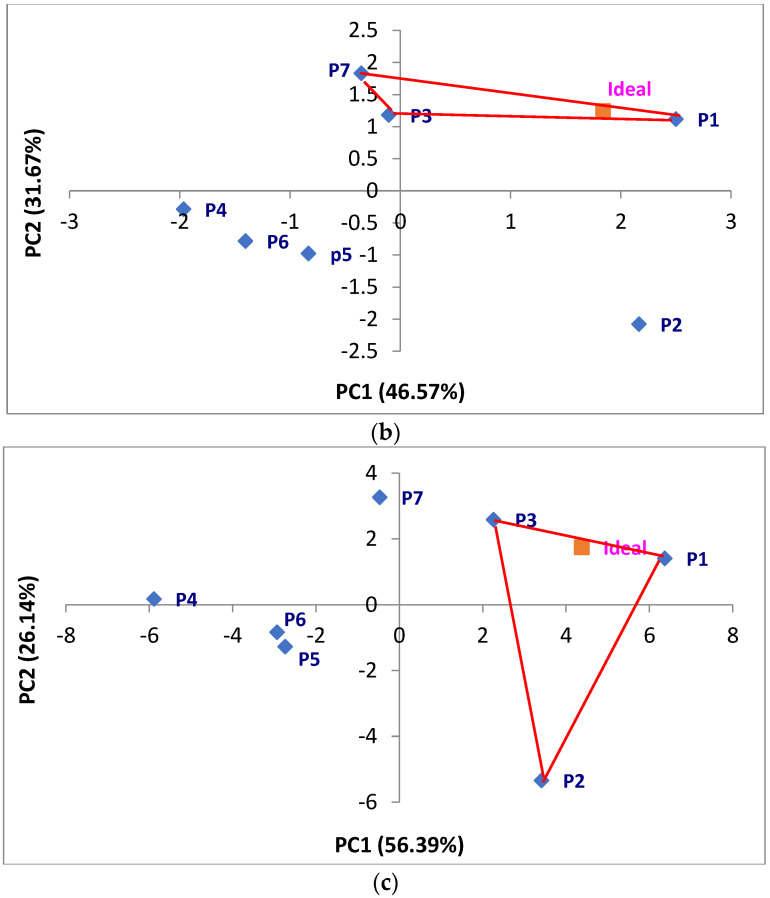
Configuration of the ideal products using the sensory data of (**a**) descriptive analysis, (**b**) rate all that apply, and (**c**) intensity scales based on RSIPM.

The optimization of apple juices using DA sensory data within the RSIPM framework is illustrated in Figure 1a.

Given that RSIPM is a type of EPM based on sensory characteristics, a sensory space was first created based on DA data. Following Danzart’s, the position of the ideal product was determined by regressing consumer preference data onto this sensory space [4]. The ideal product was contained within a three − product triangle, allowing for the calculation of the optimal ratio using the barycenter method. The optimal ratio was calculated by applying the coordinates of the three products located close to the ideal product in the barycenter formula. Consequently, the ideal product was located close to P1, P2, and P3, and the optimal ratios calculated were 36.3% for P1, 9.4% for P2, and 54.3% for P3.

#### 3.4.2. RATA Data

The optimization of apple juices using RATA sensory data within the RSIPM framework is illustrated in Figure 1b. As previously described, the RSIPM created a sensory space based on RATA data, and the ideal product location was selected by regressing consumer preference data according to Danzart’s method [4]. The RSIPM results using RATA data also identified the ideal product contained within a three-product triangle, allowing for the calculation of the optimal ratio using the barycenter method. Consequently, the ideal product was located close to P1, P3, and P7, and the optimal ratios were 76.2% for P1, 6.8% for P3, and 17.0% for P7.

#### 3.4.3. IS Data

The results of the optimization of apple juices using IS sensory data within the RSIPM framework are represented in Figure 1c. The RSIPM created a sensory space based on IS data, followed by the determination of the ideal product location through regression of consumer preference data using Danzart’s method [4]. RSIPM analysis using IS also indicated that the ideal product was contained within a three-product triangle, allowing for the calculation of the optimal ratio using the barycenter method. The ideal product was located close to P1, P2, and P3, and the optimal ratios were 50.6% for P1, 3.1% for P2, and 46.3% for P3.

### 3.5. Comparison of Methodologies

Correlation (r) and regression vector (Rv) coefficients were calculated to assess the level of similarity among the three different optimization methods. Correlation analysis is employed to examine the relationship between two or more quantitative variables based on their linear association, yielding an r-value ranging from –1 to 1. An r value of 0.5 suggests a weak positive relationship, whereas 1 indicates a perfect positive relationship. The Rv coefficient was calculated to determine the similarity between two sets of variables measured from the same sample. This value is similar to the goodness-of-fit measure (R^2^) in regression analysis, ranging from 0 to 1, with values closer to 1 indicating higher similarity [34].

**Figure 2:**
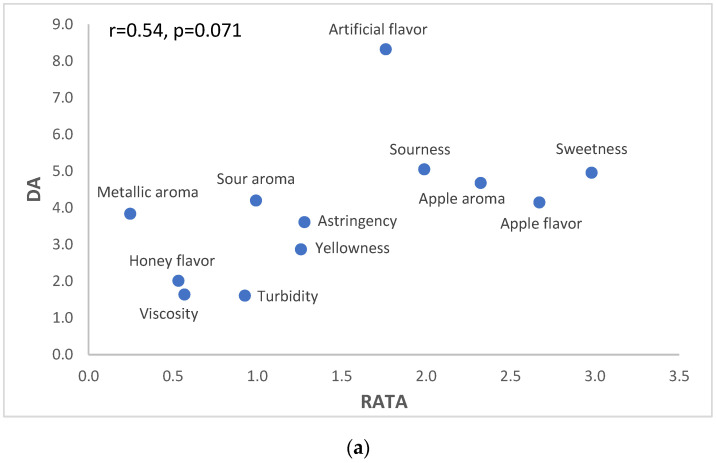

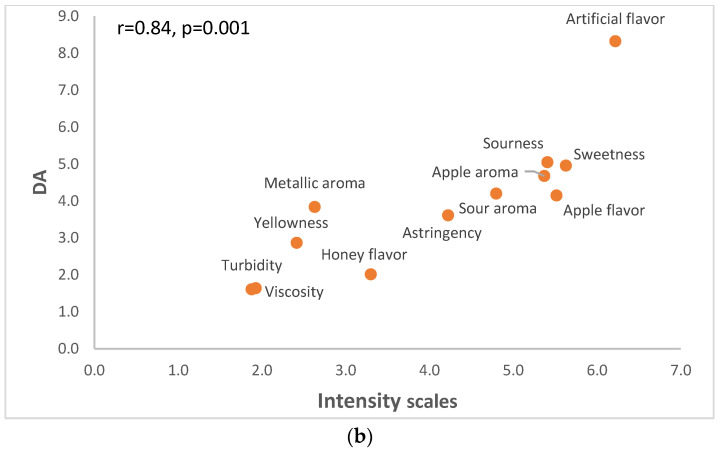
Correlation analysis plot for the ideal sensory profiles of descriptive analysis (DA) and two consumer-based methods: (**a**) DA-RATA; (**b**) DA-intensity scales.

The correlation analysis results of the sensory profiles for the ideal products optimized using DA and two consumer-based methods are presented in Figure 2.

The r value between DA and RATA was 0.54 (*p* = 0.071), and the intensity was 0.84 (*p* = 0.001). The ideal products of DA and RATA showed a relatively weak positive correlation, which can be attributed to potential scaling differences. The DA scale ranges from 0 to 15 points, whereas the RATA uses a narrower scale (0–5) intensity assessment. This disparity in scale granularity may lead to a dumping effect, potentially limiting the discriminatory power of RATA compared to that of DA [35]. Therefore, the observed low r-value may be attributed to the limited range of scales used, complicating the assessment of similarity owing to the narrow interval between intensity differences. In addition, the level of difficulty in identifying “artificial flavor” may have also contributed to the low correlation coefficient. In the correlation analysis plot (Figure 2), the ideal products of RATA and DA showed similar “sweetness” intensity; however, “artificial flavor” appeared distant from the expected relationship trend line. This divergence can be attributed to the inherent difficulty the consumers might have experienced in distinguishing between “sweetness” and “artificial flavor,” given that sweeteners also produce a sweet sensation. Therefore, these factors also influenced the correlation coefficient of the ideal sensory profiles derived from the optimal ratio analysis.

The Rv coefficients of RSIPM results calculated between DA and each of the consumer-based methods are calculated. The coefficients indicated a high degree of similarity between DA and both RATA (0.92) and IS (0.91). This strong similarity suggests that, similar to DA, both RATA and IS quantified the given sensory characteristics using the same preselected attributes. Ghanbari et al. [36] also reported that CATA demonstrated a high similarity with DA because of its use of the same preselected attribute list as DA. Although both RATA and IS aim to quantify the sensory characteristics of a product, they differ in approach. RATA offers advantages in terms of speed and cognitive ease, comparable to the CATA format, while also reducing respondent burden [37]. RATA effectively distinguishes product differences, particularly for well-known characteristics, such as basic taste or appearance-related characteristics, closely resembling DA. However, similarity tends to be lower for characteristics perceived as complex by consumers [35]. In this study, the sensory evaluation of apple juice involved 12 attributes encompassing appearance, basic taste, flavor, and mouthfeel. Among them, characteristics such as “metallic aroma” and “astringency” are expected to be difficult to detect [38]. However, consumers detected significant differences across all evaluated characteristics. These results suggest that the characteristics of commercial apple juices were relatively comprehensible and evaluable by consumers, contributing to the observed high similarity with DA results.

The line scale used in IS offers the advantage of dissociation from numerical ratings and verbal responses, thereby minimizing the potential bias associated with consumers liking or disliking specific numbers or expressions [39]. Furthermore, it provides flexibility with various positions along the length of the line to express the intensity of a sensory characteristic [40], enabling consumers to measure intensity across various ranges. However, the IS evaluation process requires assessing all given characteristics, which may become tedious for consumers, particularly when dealing with numerous characteristics [39]. Although the number of characteristics was limited to 12 in this study, an increase in the number of characteristics may amplify consumer fatigue and, consequently, reduce similarity.

In conclusion, while both the RATA and IS showed high similarity as alternatives to DA in the EPM, the product type and sensory characteristics should be considered when selecting a consumer-based profiling method. Furthermore, to develop an optimized product through the EPM, applying an appropriate consumer-based profiling method, either RATA or IS, is essential.

### 3.6. Validation Test

An additional 80 consumers were involved in the validation test to assess potential differences in preference among the ideal products obtained using the optimal ratios from three optimization methods. Lovely and Meullenet [1] highlighted that consumers in the validation and original tests may have different ideal points. Therefore, the involvement of different consumers in both tests could be a drawback, potentially affecting the validation results. However, recruiting an additional 80 consumers was deemed beneficial in examining how optimization outcomes from an initial group of 160 consumers influenced other consumers. Consumer perceptions of ideal products are inherently subjective and vary based on individual preferences and needs [41]. Understanding this diversity is important for successful product development and marketing. Therefore, in this study, the validation test involved a separate cohort of 80 consumers, different from those in the initial consumer tests.

OL scores were assessed for ideal products using DA and two consumer-based profiling datasets (i.e., RATA and IS), as well as two highly preferred products (P1 and P6) from previous consumer tests. No significant difference in OL values, except for P6 (the most preferred product), was observed among all consumers (Table 6).

The OL scores for ideal products were 6.7 for IS, 6.3 for DA, and 6.2 for RATA. Notably, the OL score for P6 was 4.9, indicating a significantly lower OL than that of the ideal products. The Friedman test results for the ranking methods are also presented in Table 6. A ranking score close to 1 indicates strong consumer preference. The mean rank scores for the ideal products were 2.4 for IS, 2.8 for DA, and 3.0 for RATA. Similar to the 9-point scale consumer liking test, no significant differences were observed among the three ideal products. However, P6 exhibited a significantly lower ranking than the other samples.

These results indicate that all ideal products were superior choices compared to the products with high OL from the initial tests. Conducting validation studies on these ideal products is a logical approach to evaluate and compare method effectiveness [1]. Therefore, these results demonstrate the effectiveness of the three optimization methods employed and the applicability of replacing DA in EPM with two consumer-based profiling methods.

## 4. Conclusions

The main advantage of using consumer-based methods with a consumer liking test is that it allows for the assessment of both consumer liking for the tested products and the intensity of products’ sensory characteristics, without the need for DA. The use of sensory profiling obtained from trained panelists in EPM plays an important role in product optimization. However, conventional DA demands significant time and effort to yield reliable results. Although significant progress has been made in using consumer-based profiling as an alternative to DA, its research extension to identifying optimal products with ideal sensory characteristics remains limited. The current study demonstrated that both RATA and IS exhibited potential as replacements for DA in identifying optimal products, as evidenced by their ability to generate ideal sensory profiles (within the RSIPM framework) equivalent to those derived from DA. Since RATA or IS can be concurrently performed when evaluating the liking of products, it would be highly beneficial to identify group ideals and their sensory characteristics through a single consumer testing session. This approach will significantly reduce the time required for making important decisions regarding product development or enhancement. Future research should explore the application of consumer-based profiling methods that directly reflect consumer opinions, such as free listing or flash profile, which have recently gained increasing attention.

## Figures and Tables

**Table 1:** Product description of seven apple juice products.

Samples	Ingredients
P1	Water, high-fructose corn syrup, apple concentrate 1% (juice 10%), citric acid, synthetic flavoring agent (apple aroma), trisodium citrate, enzymatically modified stevia, vitamin C
P2	Water, sugar, apple concentrate 1.1% (solids 70%, juice 8%), apple paste 0.6% (juice 2%), pectin, citric acid, skim milk powder 0.1%, sodium citrate, synthetic flavoring agent (apple aroma), thaumatin, enzymatically modified stevia, dextrin
P3	Water, apple concentrate 14.286% (solids 70%), synthetic flavoring agent (apple aroma), citric acid, vitamin C, DL-malic acid, steviol glycoside
P4	Water, apple concentrate 25% (solids 48%), citric acid, vitamin C, synthetic flavoring agent (apple aroma)
P5	Water, apple concentrate 25%, apple puree 1%, synthetic flavoring agent, natural flavoring agent, vitamin C
P6	Water, apple concentrate 25% (solids 50%), natural apple aroma
P7	Apple juice 100%

**Table 2:** Lexicon for apple juice products.

Attribute		Definition	Reference (Intensity)
Appearance	Yellowness	Intensity of the yellow color of apple juice	Water [0], Allulose [7] (Samyang Co., Ltd., Seoul, Republic of Korea), Olive oil [12.5] (NoBrand, Seoul, Republic of Korea)
	Turbidity	Intensity of the turbidity of apple juice	Water [0], Cool Pis pineapple flavor [13.5] (Dongwon F&B Co., Ltd., Seoul, Republic of Korea)
Aroma	Sour	Intensity of the sour aroma of apple juice	20%, 70% apple vinegar solution [5.5], [11.5] (Ottogi Co., Ltd., Seoul, Republic of Korea)
	Metallic	Intensity of the metallic aroma of apple juice	Ddaom apple juice [8.5] (Binggrae Co., Ltd., Seoul, Republic of Korea)
	Apple	Intensity of the apple aroma of apple juice	Mychew apple flavor [8] (Crown Co., Ltd., Seoul, Republic of Korea)
Flavor	Sweetness	Intensity of the sweetness of apple juice	2%, 7% sucrose solution [2], [7]
	Sourness	Intensity of the sourness of apple juice	0.05%, 0.12% citric acid solution [2], [9]
	Artificial	Intensity of the artificial flavor of apple juice	Welchs apple juice [6.5] (Nongshim Co., Ltd., Seoul, Republic of Korea), Demisoda apple flavor [13] (Donga-Otsuka Co., Ltd., Anyang city, Republic of Korea)
	Apple	Intensity of the apple flavor of apple juice	NFC apple juice [8] (NoBrand, Seoul, Republic of Korea)
	Honey	Intensity of the honey flavor of apple juice	10% honey solution [5.5] (Green food, Yongin city, Republic of Korea)
Mouth feel	Astringency	Intensity of the astringency of apple juice	50% black tea solution [2], Black tea [9] (Dongsuh Food Co., Ltd., Inchon, Republic of Korea)
	Viscosity	Intensity of the viscosity of apple juice	Water [0], Whole milk [5.5] (Seoul milk, Seoul, Republic of Korea), Yogurt [13] (Maeil Dairy Industry Co., Ltd., Seoul, Republic of Korea)

**Table 3:** Means for the sensory attributes obtained from descriptive analysis for seven apple juice products.

Attribute	P1	P2	P3	P4	P5	P6	P7
**Appearance**							
Yellowness	0.9 ± 0.3 ^1^ f ^2^	1.5 ± 0.5 e	4.4 ± 0.9 d	9.7 ± 1.0 b	4.7 ± 0.6 d	8.1 ± 1.3 c	11.2 ± 1.0 a
Turbidity	0.6 ± 0.3 e	8.2 ± 0.7 c	1.2 ± 0.4 de	11.3 ± 1.2 a	8.8 ± 1.2 c	10.1 ± 1.1 b	1.3 ± 0.5 d
**Aroma**							
Sour	4.3 ± 0.7 c	1.7 ± 1.0 e	4.6 ± 0.6 abc	5.1 ± 0.8 a	4.8 ± 0.6 ab	4.4 ± 0.6 bc	2.2 ± 0.6 d
Metallic	3.2 ± 1.0 de	2.8 ± 1.6 e	4.4 ± 1.0 bc	4.8 ± 1.1 b	6.2 ± 0.8 a	6.5 ± 0.7 a	3.8 ± 1.0 cd
Apple	5.1 ± 0.8 b	2.3 ± 0.9 c	4.8 ± 0.5 b	6.4 ± 0.7 a	5.0 ± 0.9 b	5.3 ± 0.4 b	5.9 ± 0.9 a
**Flavor**							
Sweetness	5.1 ± 1.0 bc	6.4 ± 0.8 a	4.6 ± 0.9 cd	4.3 ± 0.9 d	5.3 ± 1.3 b	6.1 ± 0.7 a	6.4 ± 0.7 a
Sourness	4.7 ± 1.5 c	1.9 ± 1.1 e	5.8 ± 1.1 b	7.9 ± 1.3 a	4.8 ± 0.9 c	3.8 ± 0.7 d	3.3 ± 0.9 d
Artificial	9.8 ± 1.2 a	8.8 ± 1.0 b	7.3 ± 1.1 c	6.6 ± 0.4 cd	6.5 ± 1.0 cd	6.4 ± 1.0 d	6.0 ± 1.6 d
Apple	3.1 ± 1.0 d	2.7 ± 0.8 d	5.1 ± 0.9 c	6.4 ± 0.9 b	6.6 ± 0.9 b	6.6 ± 0.8 b	8.6 ± 1.1 a
Honey	1.1 ± 0.4 d	2.1 ± 0.7 bc	2.6 ± 0.5 b	2.1 ± 0.7 bc	1.7 ± 0.4 c	2.3 ± 0.6 b	5.9 ± 1.0 a
**Mouthfeel**							
Astringency	3.3 ± 0.8 c	1.1 ± 0.5 e	4.2 ± 1.0 b	5.2 ± 0.9 a	3.5 ± 0.9 c	3.0 ± 0.7 c	1.7 ± 0.8 d
Viscosity	1.5 ± 0.7 c	4.2 ± 0.7 a	1.3 ± 0.3 c	3.5 ± 0.7 b	3.2 ± 0.6 b	3.5 ± 0.6 b	1.5 ± 0.6 c

^1^ Standard deviation. ^2^ Means with the same letter in the same row are not significantly different (*p* < 0.05).

**Table 4:** Means for the sensory attributes obtained from rate all that apply for seven apple juice products.

Attribute	P1	P2	P3	P4	P5	P6	P7
**Appearance**							
Yellowness	0.6 ± 0.5 ^1^ c ^2^	0.9 ± 0.8 c	3.0 ± 1.2 b	4.0 ± 1.3 a	2.8 ± 0.8 b	3.6 ± 1.1 a	3.7 ± 1.0 a
Turbidity	0.9 ± 1.3 b	3.4 ± 1.3 a	1.5 ± 1.7 b	3.5 ± 2.0 a	3.4 ± 1.4 a	3.7 ± 1.5 a	0.8 ± 0.9 b
**Aroma**							
Sour	1.0 ± 1.3 ab	0.4 ± 0.8 b	1.4 ± 1.6 a	1.5 ± 1.7 a	1.3 ± 1.6 a	1.1 ± 1.5 a	0.9 ± 1.3 ab
Metallic	0.1 ± 0.4 d	0.4 ± 1.0 cd	0.6 ± 1.2 cd	0.9 ± 1.4 c	2.0 ± 1.9 a	1.5 ± 1.7 ab	0.9 ± 1.5 bc
Apple	2.4 ± 1.4 a	1.1 ± 1.3 b	2.3 ± 1.4 a	2.4 ± 1.6 a	2.0 ± 1.4 a	2.4 ± 1.6 a	1.8 ± 1.7 ab
**Flavor**							
Sweetness	3.1 ± 1.5 a	2.8 ± 1.6 ab	2.3 ± 1.5 b	2.1 ± 1.7 b	2.6 ± 1.6 ab	2.8 ± 1.6 ab	2.5 ± 1.7 ab
Sourness	2.0 ± 1.8 b	0.7 ± 1.1 c	2.4 ± 1.7 ab	2.9 ± 1.5 a	2.0 ± 1.7 b	1.7 ± 1.5 b	1.7 ± 1.7 b
Artificial	1.9 ± 1.9 ab	2.5 ± 2.0 a	1.5 ± 1.9 b	1.5 ± 1.7 b	1.8 ± 1.9 ab	1.4 ± 1.7 b	1.4 ± 1.6 b
Apple	2.8 ± 1.5 ab	1.3 ± 1.4 d	2.1 ± 1.5 bc	2.0 ± 1.6 cd	2.4 ± 1.4 abc	2.9 ± 1.4 a	2.5 ± 1.7 abc
Honey	0.2 ± 0.6 c	0.7 ± 1.3 bc	0.6 ± 1.2 bc	0.4 ± 1.2 bc	0.6 ± 1.2 bc	0.9 ± 1.4 b	2.1 ± 1.9 a
**Mouthfeel**							
Astringency	1.3 ± 1.6 b	0.4 ± 0.8 c	1.3 ± 1.6 b	2.0 ± 1.7 a	1.6 ± 1.6 ab	1.4 ± 1.5 ab	1.3 ± 1.5 ab
Viscosity	0.5 ± 0.8 b	1.3 ± 1.6 a	0.6 ± 1.2 ab	1.2 ± 1.6 a	1.3 ± 1.6 b	1.2 ± 1.7 a	0.9 ± 1.3 ab

^1^ Standard deviation. ^2^ Means with the same letter in the same row are not significantly different (*p* < 0.05).

**Table 5:** Means for the sensory attributes obtained from intensity scales for seven apple juice samples.

Attribute	P1	P2	P3	P4	P5	P6	P7
**Appearance**							
Yellowness	0.2 ± 0.4 ^1^ f ^2^	1.5 ± 1.3 e	4.9 ± 2.1 d	9.0 ± 1.4 a	6.1 ± 2.0 c	6.6 ± 1.7 c	8.0 ± 1.6 b
Turbidity	1.5 ± 3.3 c	7.0 ± 2.2 b	1.9 ± 2.4 c	8.4 ± 2.7 a	7.4 ± 2.0 ab	7.2 ± 2.3 b	2.0 ± 2.4 c
**Aroma**							
Sour	5.0 ± 2.9 ab	2.1 ± 2.4 c	4.8 ± 3.0 ab	5.6 ± 2.9 a	4.9 ± 2.6 ab	4.5 ± 2.8 ab	3.8 ± 3.1 b
Metallic	2.3 ± 2.3 c	2.6 ± 2.8 c	3.0 ± 2.6 bc	4.6 ± 2.9 a	5.0 ± 3.0 a	5.0 ± 3.3 a	4.2 ± 3.1 ab
Apple	5.4 ± 2.8 a	3.5 ± 2.7 b	5.4 ± 2.2 a	5.8 ± 2.7 a	5.5 ± 2.7 a	5.7 ± 2.6 a	5.3 ± 3.1 a
**Flavor**							
Sweetness	5.3 ± 2.7 b	6.4 ± 2.5 ab	6.0 ± 5.0 ab	5.5 ± 2.8 ab	6.5 ± 2.3 ab	6.8 ± 2.1 a	6.3 ± 2.5 ab
Sourness	6.2 ± 2.8 a	2.2 ± 1.9 c	4.8 ± 2.6 b	7.0 ± 2.4 a	4.5 ± 2.7 b	4.6 ± 2.4 b	4.0 ± 2.6 b
Artificial	6.8 ± 2.4 a	6.8 ± 2.6 a	5.6 ± 2.8 ab	6.0 ± 2.6 ab	5.4 ± 2.8 b	5.3 ± 2.7 b	5.1 ± 2.9 b
Apple	5.3 ± 2.5 bc	4.2 ± 2.7 c	5.8 ± 2.2 ab	6.8 ± 4.1 a	6.5 ± 2.2 ab	6.5 ± 2.2 ab	5.9 ± 2.7 ab
Honey	2.4 ± 1.9 d	4.6 ± 2.9 bc	4.2 ± 2.8 bc	3.5 ± 2.6 cd	4.8 ± 2.7 bc	4.9 ± 2.5 b	6.2 ± 3.1 a
**Mouthfeel**							
Astringency	4.2 ± 3.1 b	2.0 ± 2.4 c	4.4 ± 3.0 b	6.2 ± 2.6 a	4.6 ± 3.0 b	5.0 ± 3.0 ab	4.3 ± 3.0 b
Viscosity	1.5 ± 1.7 e	3.3 ± 2.6 cd	2.3 ± 2.0 de	5.4 ± 2.6 a	4.5 ± 2.8 ab	4.1 ± 2.7 bc	2.8 ± 2.7 d

^1^ Standard deviation. ^2^ Means with the same letter in the same row are not significantly different (*p* < 0.05).

**Table 6:** Overall liking and rank scores for three ideal products based on RSIPM and the product that scored highest in OL in the initial study (validation test).

Products	Overall Liking	Rank Scores ^1^
DA ideal product	6.3 ± 1.7 ^2^ a ^3^	2.8 ± 1.2 a ^3^
RATA ideal product	6.2 ± 1.6 a	3.0 ± 1.3 a
Intensity scale ideal product	6.7 ± 1.6 a	2.4 ± 1.1 a
P1	6.2 ± 1.8 a	2.9 ± 1.4 a
P6	4.9 ± 2.4 b	3.9 ± 1.5 b

^1^ Rank scales (1 = most preferable, 3 = medium, 5 = least preferable). ^2^ Standard deviation. ^3^ Means with the same letter in the column are not significantly different (*p* < 0.05). RSIPM = response surface ideal point mapping. DA = descriptive analysis.

## Data Availability

The data presented in this study are available on request from the corresponding author. The data are not publicly available due to privacy restrictions.
